# Anomalous Discharge of Endogenous Gas at Lavinio (Rome, Italy) and the Lethal Accident of 5 September 2011

**DOI:** 10.1029/2019GH000211

**Published:** 2019-12-03

**Authors:** F. Barberi, M. L. Carapezza, L. Tarchini, M. Ranaldi, T. Ricci, A. Gattuso

**Affiliations:** ^1^ Sezione Roma1 INGV–Istituto Nazionale di Geofisica e Vulcanologia Rome Italy; ^2^ Sezione di Palermo, INGV–Istituto Nazionale di Geofisica e Vulcanologia Italy

**Keywords:** hazard from endogenous gas emission, lethal gas accident, Lavinio; Rome, Italy

## Abstract

The Rome region contains several sites where endogenous gas is brought to the surface through deep reaching faults, creating locally hazardous conditions for people and animals. Lavinio is a touristic borough of Anzio (Rome Capital Metropolitan City) that hosts a country club with a swimming pool and an adjacent basement balance tank. In early September 2011, the pool and the tank had been emptied for cleaning. On 5 September, four men descended into the tank and immediately lost consciousness. On 12 August 2012, after a long coma the first person died, the second one reported permanent damage to his central nervous system, and the other two men recovered completely. Detailed geochemical investigations show that the site is affected by a huge release of endogenous gas (CO_2_ ≈ 96 vol.% and H_2_S ≈ 4 vol.%). High soil CO_2_ and H_2_S flux values were measured near the pool (up to 898 and 7.155 g·m^−2^·day^−1^, respectively), and a high CO_2_ concentration (23–25 vol.%) was found at 50–70 cm depth in the soil. We were able to demonstrate that gas had been transported into the balance tank from the swimming pool through two hubs connected to the lateral overflow channels of the pool. We show also that the time before the accident (60 hr), during which the balance tank had remained closed to external air, had been largely sufficient to reach indoor nearly lethal conditions (oxygen deficiency and high concentration of both CO_2_ and H_2_S).

## Key Points

- In volcanic and geothermal areas, the emission of deep gas locally creates hazardous conditions for people and animals, as at Lavinio
- Lavinio country club hosts a swimming pool with a basement tank, where an accident occurred on 5 September 2011 causing the death of a man
- Geochemical studies show that the man inhaled a nearly lethal CO_2_‐H_2_S‐rich air mixture, transported from the pool into the basement tank

## 1 Introduction

An enormous quantity of volcanic gases, including carbon dioxide (CO_2_), sulfur dioxide (SO_2_), and hydrogen sulfide (H_2_S), is emitted during volcanic eruptions and also during passive intereruptive degassing of open conduit volcanoes, such as Etna, Stromboli, and Kilauea. In volcanic and geothermal areas, gas is released from fumarolic vents or diffusively through the soil. Besides the dramatic direct effects on human life caused by the volcanic gases emitted during explosive eruptions, health hazards are associated also with low‐temperature gas vents or diffuse soil emissions (Aramaki et al., 1994; Hansell & Oppenheimer, 2005). The largest number of casualties have been produced by CO_2_ clouds released from two crater lakes of Cameroon, Lake Monoun in 1984 with 37 victims (Sigurdsson et al., 1987) and Lake Nyos in 1986 with 1,700 deaths (Kling et al., 1987) and by the 1979 phreatic explosions of Dieng Plateau, Indonesia, with 139 deaths (Le Guern et al., 1982). Several deaths and adverse health effects caused worldwide, particularly in Italy, Japan, and New Zealand, by inhalation of CO_2_ or H_2_S in volcanic and geothermal areas are reported in the review of Hansell and Oppenheimer (2005). This includes a man, as well as cows and sheep, killed by gas at Cava dei Selci, a gas emitting site of Albani Hills in the Rome region (Carapezza et al., 2003).

**Figure 1:**
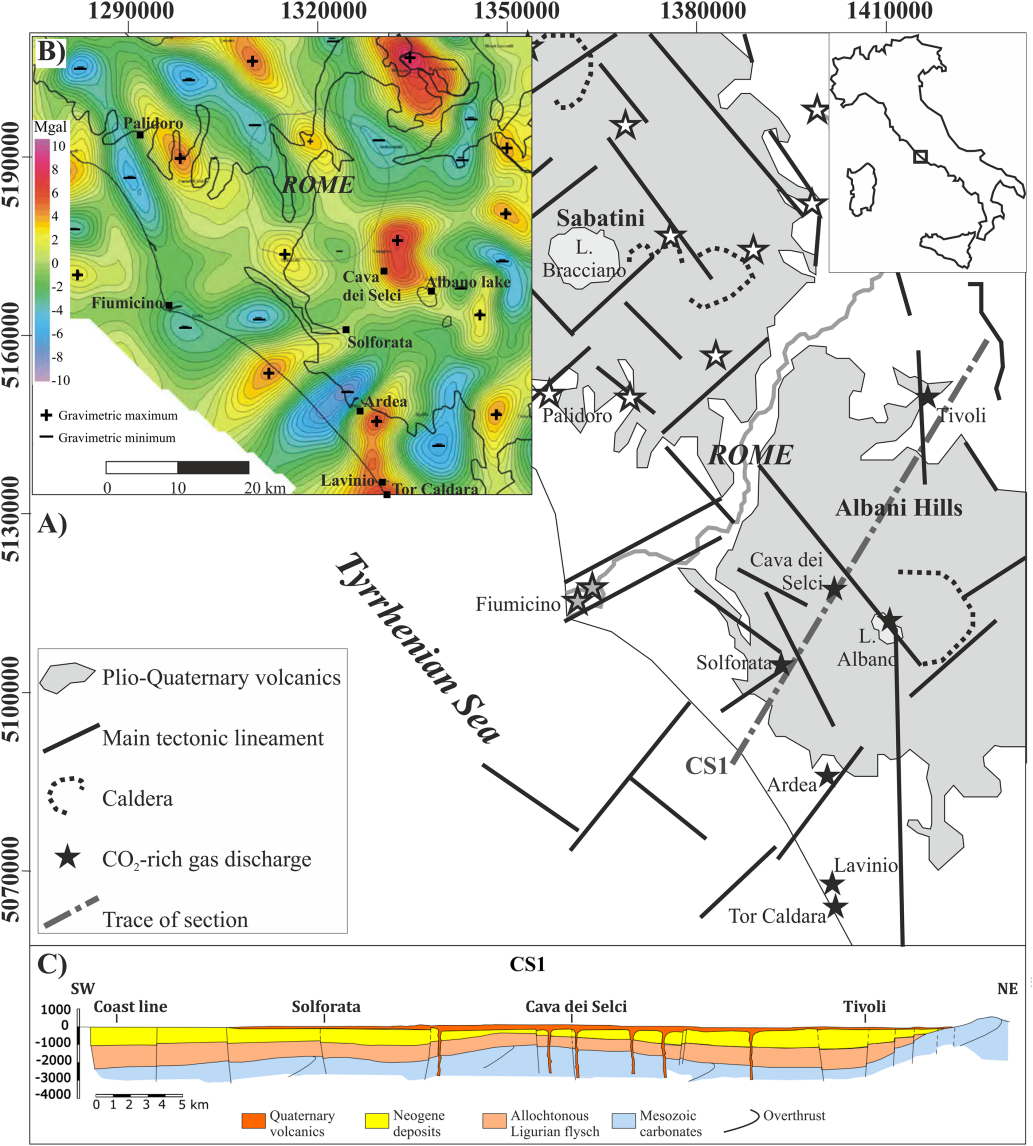
(a) Main tectonic lineaments of the Rome region (modified after Acocella & Funiciello, 2006) and location of CO_2_‐rich gas discharges from Albani Hills (black stars), from Mts. Sabatini (white stars) and from Fiumicino (gray stars). (b) High‐pass map of Bouguer gravity anomalies (after Cesi et al., 2008), with location of the main gas discharge zones (black squares). (c) Geological structure of the Rome area illustrated by a NE‐SW geological profile passing through Cava dei Selci and Solforata gas‐discharging zones of Albani Hills (CS1 profile obtained from geological and geophysical data and deep exploratory geothermal wells, modified after Enel et al., 1987).

The Rome region, located in central Italy between two Quaternary volcanoes (Mts. Sabatini to the NW and Albani Hills to the SE), actually contains several zones characterized by anomalous and hazardous discharge of endogenous gas (mostly CO_2_ but with a significant H_2_S content up to 6.3 vol.%; Carapezza et al., 2019; Figure 1a).

Lavinio is a touristic borough of Anzio Municipality, which in turn belongs to the Rome Capital Metropolitan City. Lavinio extends for nearly 4 km along the Tyrrhenian Coast to the NW of Tor Caldara, a regional reserve hosting the southwestern most site of endogenous gas discharge of Albani Hills (Figure 1a).

An extensional geochemical study, including soil CO_2_ and H_2_S flux surveys, air gas concentration measurements, and chemical and isotopic characterizations of the emitted gas (summarized in Carapezza et al., 2019), showed that the Lavinio‐Tor Caldara zone is interested by an anomalous and locally hazardous discharge of CO_2_ and H_2_S of deep provenance.

**Figure 2:**
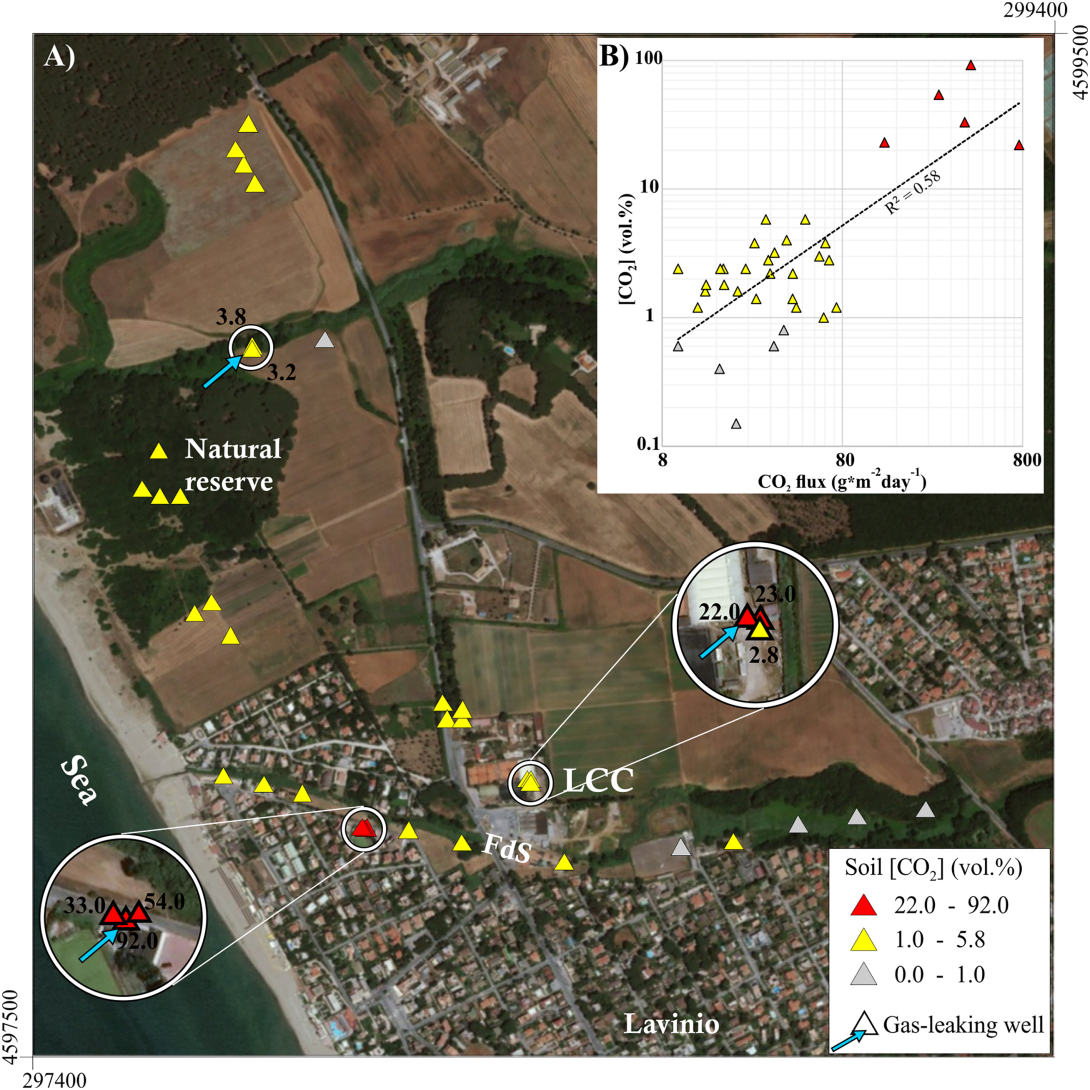
(a) Soil CO_2_ concentration at 50‐cm depth in the Lavinio zone (July to September 2011). The highest values (red triangles) have been found near old gas leaking wells at Lavinio Country Club (LCC) and in the nearby Fosso dello Schiavo River (FdS in the figure). (b) Soil CO_2_ concentration compared with soil CO_2_ flux in the same points (log scale).

The zone hosts the Lavinio Country Club (hereafter LCC), a sporting club where tennis and swimming are practiced, that is located at about 2.5 km NW of Tor Caldara and 600 m from the Tyrrhenian Sea (location in Figure 2). Here, on 5 September 2011, an accident occurred to four workers, having lethal consequences for a man. A detailed geochemical investigation was carried out immediately after the accident, aimed at clarifying its causes.

## 2 Geological and Geochemical Outlines

As shown in Figure 1b, all discharges of endogenous gas of the Rome region are located above structural highs of the buried dense Mesozoic carbonate basement, revealed by positive gravimetric anomalies and frequently ascertained by deep geothermal wells (Cataldi et al., 1995). These carbonates host the most important regional aquifer, where gas rising from depth dissolves and accumulates. The carbonate reservoir is covered by an impervious cap of allocthonous flysch deposits (Ligurian units), in turn covered by Neogene sediments and by Quaternary volcanic products (see the geological cross‐section of Figure 1c). Also, Lavinio zone is located on a structural high of the carbonate basement, directed N‐S and NNE‐SSW toward the Albano Crater Lake, that is at 24‐km distance (Figure 1b).

Gas escapes from the reservoir to the surface through deep reaching faults and may dissolve into and pressurize shallower confined aquifers that frequently produce hazardous gas blowouts when reached by wells (Carapezza et al., 2019 and references therein).

Albani Hills is a complex volcano belonging to the Roman potassic comagmatic province (Washington, 1906) whose eruptive activity initiated about 600 ka BP and lasted up to 5.8 ka. The recent most eruptive vent is hosted within the summit Albano Crater Lake from where recent dangerous lahars have been generated by water overflow, producing impervious superficial deposits. In the fourth century BCE, Romans excavated a drainage tunnel in the crater wall to keep low the lake water level: this is the first risk prevention work of human mankind (Carapezza et al., 2010; Funiciello et al., 2010 and references therein).

In zones where the surface impervious cover has been removed by quarrying or mining excavations (e.g., Cava dei Selci, Solforata, Tor Caldara; location in Figure 1a), gas discharges freely at the surface creating hazardous conditions. One man and animals of even large size (cows and sheep) have been killed by the gas at Cava dei Selci (Carapezza et al., 2003) and carcasses of dogs, cats, and hedgehogs are commonly found in all the gas discharging sites (Carapezza et al., 2019).

With a concentration ranging from 93 to 99 vol.%, CO_2_ is the dominant gas of the Rome region discharges (Carapezza et al., 2012, 2019). In the Lavinio‐Tor Caldara zone, the second most abundant gas is H_2_S with 4‐6 vol.% content, whereas in the other gas discharging sites indicated in Figure 1, the second most abundant gas is N_2_. The helium isotopic composition, that is, the ^3^He/^4^He ratio (*R*) expressed with respect to the same ratio in air (Ra), of these gases ranges from 0.65 to 1.90 (Carapezza et al., 2012). Although being much lower than in the typical mantle (*R*/Ra ≈ 8.0; Marty & Jambon, 1987), it is similar to the *R*/Ra found in the fluid inclusions of phenocrysts of Albani Hills volcanic rocks (Martelli et al., 2004). Therefore, helium isotopic data suggest an origin of the gas from a deep magma source affected by crustal contamination during its primary generation in a subduction process (Carapezza & Tarchini, 2007).

## 3 Materials and Methods for Gas Investigations

The CO_2_ and H_2_S soil fluxes have been measured with the accumulation chamber method described by Chiodini et al. (1998) by a portable fluxmeter manufactured by West Systems. The device was equipped with an infrared Licor‐Li820 detector for CO_2_ (range = 0–2 vol.%) and with a Tox‐05 detector for H_2_S (range = 0–20 ppm).

The CO_2_ soil flux map of Figure 4a was produced in Golden Software Surfer© by ordinary kriging geostatistical interpolation (quadratic model with scale = 0.68 and length = 34.0; Nugget effect error variance = 0.32). The CO_2_ and H_2_S soil flux data of Figures 4b, 4c, and 4d were plotted as classed post maps in Golden Software Surfer©.

The soil CO_2_ and H_2_S flux classes (statistical levels) were obtained by graphical investigation of the normal probability plot of the flux data (Yusta et al., 1998). Soil CO_2_ fluxes can be produced both by biological processes (the so‐called “soil respiration”) and by endogenous (volcanic and geothermal) ones. Typically, biological fluxes are low and up to a few tens of g*m^‐2^ day^‐1^, while endogenous fluxes can be as high as thousands of g·m^−2^·day^−1^. In the Tyrrhenian side of central Italy, soil respiration can account for soil CO_2_ fluxes lower than 30 g·m^−2^·day^−1^ in late summer (Rey et al., 2002). The main biological sources of H_2_S are the decay of organic sulfur and the activity of sulfate‐reducing bacteria (Riemenschneider et al., 2005). Voltaggio and Spadoni (2009) estimated the soil H_2_S flux background at Solforata gas emission site on Albani Hill from 10^−8^ to 10^−6^ kg/m^2^·day, so all our measured flux values (minimum = 0.01 g/m^2^·day, which is the device detection limit) can be attributed to an endogenous source.

Soil gas concentration was measured using a steel probe inserted in the ground at 50‐cm depth and connected to the device by a silicon tube. The air and soil concentration of CO_2_, H_2_S, and O_2_ were measured with portable multigas devices (Draeger X‐am 7000), used in both passive and active modes respectively, equipped with an infrared CO_2_ detector (0–100 vol.%) and electrochemical cells for both H_2_S (0–500 and 0–1,000 ppm) and O_2_ (0–25 vol.%). Draeger tubes (range = 0.2–7 vol.%) were also used to have a quick estimate of the H_2_S air concentration directly in the field, in sites where Draeger X‐am 7000 had reached its upper detection limit. Continuous air gas concentration monitoring has been carried out with the same devices in passive mode.

## 4 Hazardous Air Concentration Thresholds for CO_2_ and H_2_S

The exposure limits for human health of CO_2_ and H_2_S air volume concentration established in the occupational guidelines (NIOSH, 2007) are the following:

- Time‐weighted average (TWA), 8 hr: CO_2_ = 0.5% and H_2_S = 10 ppm.
- Short‐term exposure limit (STEL), 15 min: CO_2_ = 3.0% and H_2_S = 15 ppm.

The potentially lethal air concentration threshold is 8 vol.% for CO_2_ and 250 ppm for H_2_S (Carapezza et al., 2011 and references therein). The National Institute for Occupational Safety and Health level of H_2_S that interferes with the ability to escape (immediately dangerous to life and health, IDLH) is 100 ppm (OSHA, 2019). A H_2_S concentration of 500‐700 ppm produces staggering, collapse in 5 minutes, serious damage to the eyes in 30 minutes, death after 30‐60 minutes (OSHA, 2019).

A high CO_2_ air concentration implies a correspondent reduction in the oxygen air content. At sea level and dry air, respiratory protection is needed for O_2_ concentrations below 16 vol.% and the oxygen level immediately dangerous to life and health (IDHL) is 12.5 vol.% (Mc Manus, 2009).

## 5 Soil CO_2_ Concentration and Flux at Lavinio

In July and August 2011, we measured the CO_2_ concentration at 50‐cm depth in the soil in 32 sites in the northern part of Lavinio (data in Barberi et al., 2019). Three additional sites were measured in September 2011 at LCC shortly after the 5 September accident. Results are presented in Figure 2. Only in five sites the soil CO_2_ concentration was <1 vol.%; in the majority of the points (25 measurements), it ranged 1–5.8 vol.%; and in other five points, it was comprised from 22 to 92 vol.%. The most anomalous values were all found in the proximity of old gas leaking wells, within a narrow area including either the small Fosso dello Schiavo River (with 33, 54, and 92 vol.%) and the nearby LCC site (with 22 and 23 vol.%; Figure 2a). In these two sites, also high soil CO_2_ flux values (up to 413 and 765 g·m^−2^·day^−1^, respectively) have been measured.

In the insert of Figure 2b, soil CO_2_ concentrations are compared with soil CO_2_ fluxes measured in the same points. As soil CO_2_ fluxes below 34.2 g·m^−2^·day^−1^ are considered of biogenic or mixed origin (see chapter 6.1), the corresponding soil CO_2_ concentration values up to 5.8 vol.% can equally be considered of biogenic or mixed origin. Similar soil CO_2_ concentration values (5.1–5.2 vol.%) have been found at Torre Alfina, a geothermal site of central Italy, with carbon isotopic composition of CO_2_ (δ^13^C_CO2_ = −24.2/−25.3) indicating a biogenic origin (Braun et al., 2018).

## 6 The 5 September 2011 Accident at LCC and Related Geochemical Investigations

**Figure 3:**
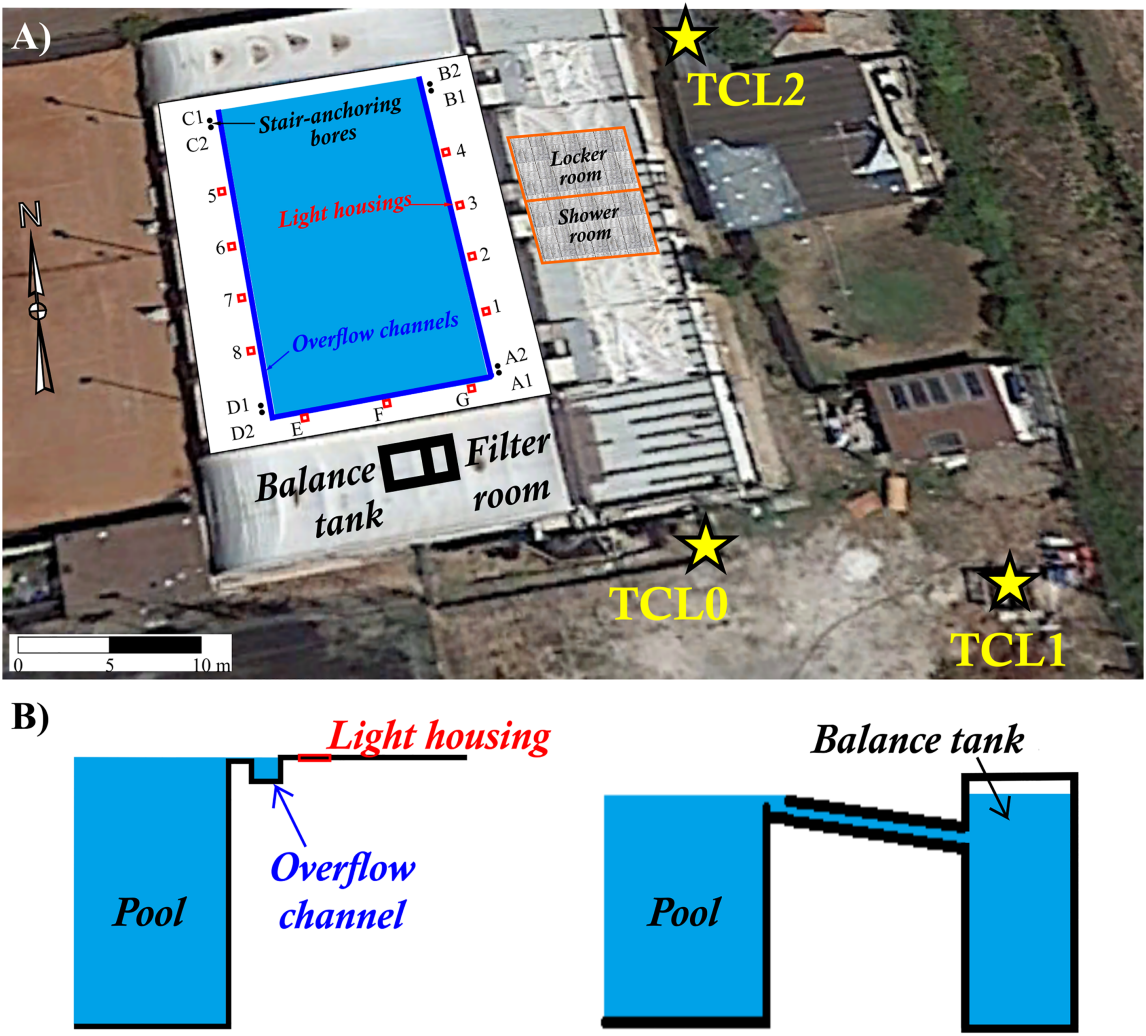
(a) Google Earth image of a sector of Lavinio Country Club with location of water wells TCL0, TCL1, TCL2 (yellow stars), swimming pool, filter room and balance tank (black frame), and children's locker and shower rooms (orange frame). Letters A1 to D2 refer to eight small holes where the pool stairs are inserted; numbers 1 to 8 and letters E, F, and G refer to the pool light housings. The small lateral channels carrying the overflow water to the balance tank are indicated by blue lines. (b) Scheme (not at scale) of the swimming pool and connected pipelines bringing water to the balance tank.

The LCC swimming pool was built in 1988 to 1989, together with two nearby basement service rooms: a pool filter room and a balance tank, where the pool overflow water was collected by means of two drainage channels located along the longer sides of the pool (Figure 3).

On 31 August 2011, the swimming pool had been emptied from water for its seasonal cleaning operations. The balance tank was emptied on 1 September and it remained closed (to the external air) from 3 to 5 September, when four men descended one after the other to clean it. The first man immediately lost consciousness and fell on the floor, and the same happened successively to the other three persons; a fifth person standing outside immediately asked for help and the fire brigade intervened promptly. It took about 45 min to remove all the men from the tank by the firefighters. In the site, there was also an ambulance, and doctors immediately provided a first aid before transferring them to the hospital. Men were all unconscious and the oldest one (60 years) was suffering a cardiac arrest. The first man (exposed for nearly 45 min) remained in coma for nearly a year and then he died. The second man (exposed for nearly 40 min), after a short period of coma, survived, but he reported permanent light damage to the central nervous system (difficulty in moving and speaking); the other two men (exposed for nearly 30 min) recovered completely.

The LCC management provided us some information on the first geological investigations carried out on the site in 2005 (16 years after the building of the swimming pool) and aimed at exploring the possibility of developing a spa in the area. The concentration of CO_2_, H_2_S, and H_2_ was measured in 10 points at 70‐cm depth in the soil. High values were found in a point located immediately south of the swimming pool: CO_2_ ≥ 25 vol.% (upper limit of the used device), H_2_S = 57 ppm, and H_2_ = 140 ppm. Here, we found 23 vol.% of CO_2_ at 50‐cm depth in September 2011. A CO_2_ concentration of 7.3 vol.% was found in 2005 (at about 1‐m depth) in a point located ~30 m to the east. In the other 2005 measures, CO_2_ concentration ranged from 0.18 to 3.4 vol.%, and H_2_S and H_2_ were absent. On May 2005, a water well was drilled at 42‐m depth (TCL1 in Figure 3). It encountered two aquifers separated by a 11.6‐m thick shale layer. The shallower aquifer, hosted in sands down to 11.4 m depth, had a temperature (*T*) of 24 °C; the second aquifer extended from 23 to 32.5 m depth, and it had a *T* = 34 °C and was hosted in volcanic ash and fractured welded tuffs. This layer contained a significant quantity of dissolved gas, as during flowing tests a free gas discharge occurred. In this gas we measured in 2011 a helium isotopic composition (*R*/Ra = 0.65) indicating a deep endogenous origin (see also Carapezza et al., 2019).

No drilling data and depth are known for TCL2 well but by a phreatimeter, we estimated a minimum depth of 10.1 m. According to the LCC manager, a third well (TCL0; location in Figure 3) was drilled in the 1970s and had been immediately cemented because it produced a strong gas emission (likely a gas blowout).

Therefore, it was already known in 2005 that LCC was a low‐temperature thermal area interested by an anomalous soil release of cold endogenous gas; however, no action was taken to protect staff and swimmers, and the pool was run normally since.

### 6.1 Soil Gas Flux Investigations

**Figure 4:**
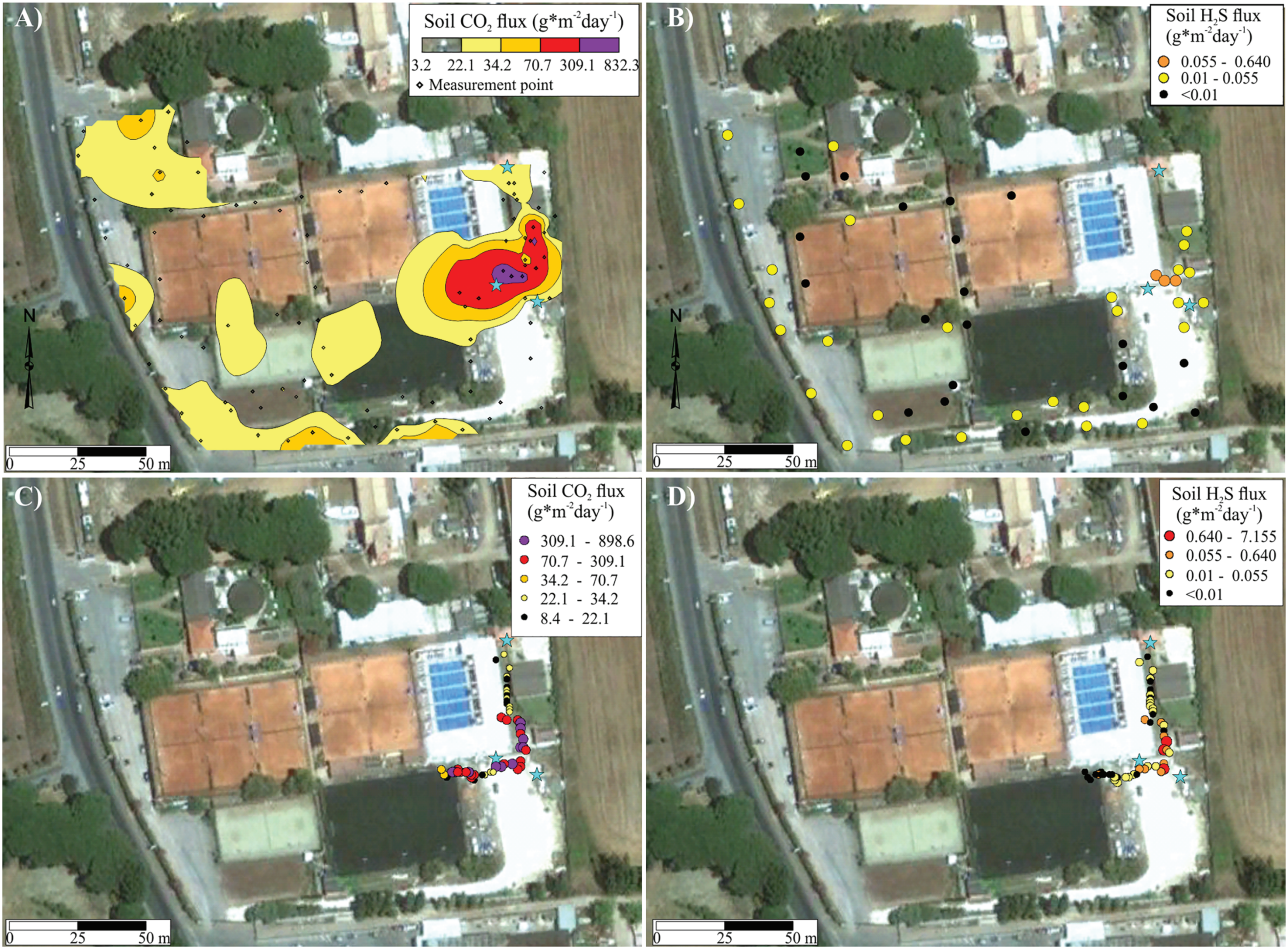
Soil flux maps of CO_2_ (a) and H_2_S (b) of Lavinio Country Club measured on 6 September 2011 with 10–13 m spacing of the measurement points. Blue stars indicate water wells TCL0, TCL1, and TCL2 (see Figure 3 for their precise location). Note that the highest soil gas anomaly is located in the proximity of well TCL0. The lowest detection limit of the H_2_S device is 0.01 g·m^−2^·day^−1^. (c) and (d) Soil CO_2_ and H_2_S flux values, with 1 m spacing, measured at Lavinio Country Club on 8 September 2011.

On 6 September 2011, a soil CO_2_ and H_2_S flux survey was carried out at LCC, with 93 and 53 measurement points, respectively, and a 10–13 m spacing (data in Barberi et al., 2019). The resulting soil CO_2_ flux map is shown in Figure 4a. The first statistical level up to 22.1 g·m^−2^·day^−1^ (black in the map) represents the background class attributed to biogenic process; the second level, flux between 22.1 and 34.2 g·m^−2^·day^−1^ (yellow in the map) likely represents a mixture of biogenic and endogenous gas. Fluxes exceeding 34.2 g·m^−2^·day^−1^ are all of endogenous origin. A strong soil gas release was observed in a small area between TCL0 and TCL1 wells, near the SE swimming pool corner (up to 832 g·m^−2^·day^−1^ for CO_2_ and 0.64 g·m^−2^·day^−1^ for H_2_S; Figures 4a and 4b). In order to investigate more in detail the anomalous zone near to the swimming pool, a denser survey with 50 new soil CO_2_ and H_2_S flux measurements with only 1 m spacing was carried out on 8 September 2011 (data in Barberi et al., 2019). The results (maximum CO_2_ flux = 899 g·m^−2^·day^−1^ and maximum H_2_S flux = 7.155 g·m^−2^·day^−1^) confirmed an anomalous gas release from the soil near the balance tank and filter room area (Figures 4c and 4d). It has to be remarked that soil H_2_S flux values between 0.640 and 7.155 g·m^−2^·day^−1^ indicate a strongly anomalous H_2_S release.

### 6.2 Outdoor CO_2_ and H_2_S Air Concentration Measurements

It has to be preliminarily noted that in this paper, the CO_2_ air concentration is reported in excess to that of the normal air (392 ppm in 2011; NOAA, 2019); this means that a 0 value corresponds to normal atmospheric air.

**Figure 5:**
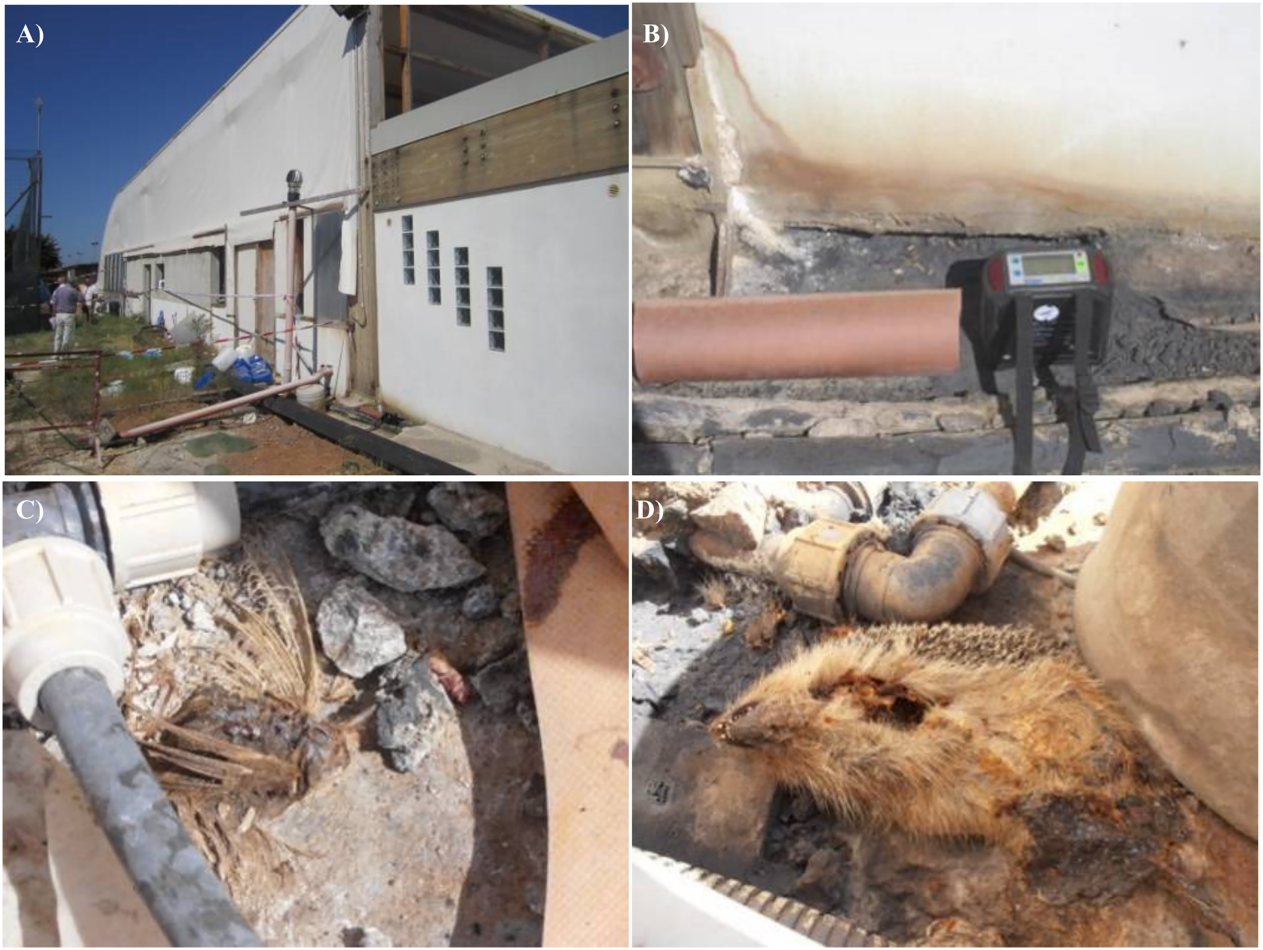
(a) The external southern wall of the technical rooms. (b) Device measuring CO_2_ and H_2_S air concentrations. (c) and (d) Evidence of alteration of the metallic parts and dead animals.

Outdoor gas concentration measurements were made at LCC from 6 to 26 September 2011. At the base of the southern external wall of the technical rooms (water balance tank and filter room; Figure 5a), we observed the presence of small dead animals (birds, reptiles, and insects), together with evidence of alteration of the metallic parts with sulfur incrustations (Figures 5c and 5d).

This is the zone where the old TLC0 well had been drilled and immediately cemented because it produced a gas blowout. Here, at a few centimeters above ground, we found the air gas concentration values reported in Table 1. In the same Table we report also the gas concentration values measured in a small runoff pit located outside the door of the filter room.

**Table 1:** Outdoor Gas Air Concentration at LCC

	6 September (no wind)	8 September (wind gusts)	26 September (no wind)
Above the old cemented TCL0 well (see Figure 6 for location)			
CO_2_ vol.%	2.4	3.6	86
H_2_S ppm	690	420	2.6 vol.%^a^
Runoff pit near the filter room			
Ground level (0 cm)			
CO_2_ vol.%	1.8	0	‐
H_2_S ppm	240	160	‐
20 cm above the water level in the pit (~80 cm below ground level)			
CO_2_ vol.%	>46^b^	>6^b^	‐
H_2_S ppm	>1000^c^	>1000^c^	‐

Abbreviation: ppm = parts per million.

a By Draeger tube.

b Device removed before signal stabilization to avoid damages by H_2_S.

c Upper limit of the used device.

**Figure 6:**
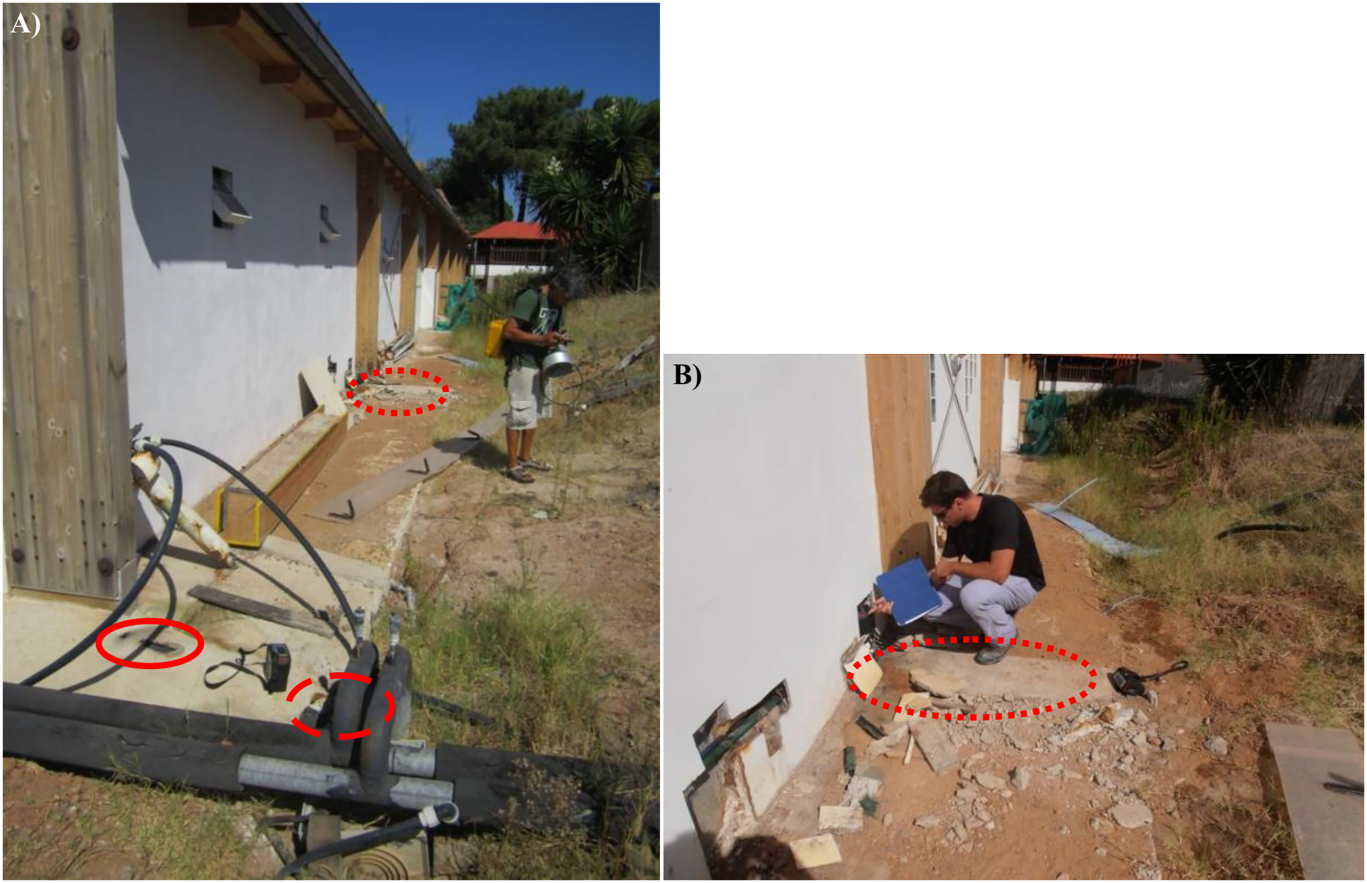
(a) SE corner and (b) eastern wall of the building hosting the service rooms of the Lavinio Country Club pool. Solid, hatched, and dotted red circles indicate the sites where outdoor gas air concentration was measured (results in Table 2).

Table 2 reports the gas air concentrations measured around the service rooms, in correspondence of small open fractures in the concrete curb bordering the building. In particular, measurements were carried out at the southeastern corner of the building hosting the service rooms (red circle in Figure 6a), at the end of the concrete curb (hatched red circle in Figure 6a), and outside the concrete curb at the base of the eastern building wall (dotted red circle in Figure 6b).

**Table 2:** Gas Air Concentrations at the SE (Figure 6a) and E (Figure 6b) Base of the Service Rooms

	6 September	8 September	26 September
Fracture in the concrete curb	(No wind)	(Wind gusts)	(No wind)
H_2_S ppm	>1000^a^	>1000^a^	‐
CO_2_ vol.%	5.8	3.6	‐
Southern concrete curb end			‐
H_2_S ppm	680	46	‐
CO_2_ vol.%	3.2	0	‐
Fracture outside the concrete curb			‐
H_2_S ppm	26	39	2.6 vol.% (by Draeger tube)
CO_2_ vol.%	0	0	86
Eastern concrete curb end			
H_2_S ppm	‐	>1000^a^	‐
CO_2_ vol.%	‐	54	‐

Abbreviation: ppm = parts per million.

a Upper limit of the used device.

The metallic casing of well TCL1 appeared altered by the long exposure to the gas; the gas concentration estimated at −1.5 m within the casing was 96 vol.% of CO_2_ and 4.2 vol.% of H_2_S (concentration values similar to the Tor Caldara gas composition; Carapezza et al., 2012, 2019).

### 6.3 Indoor Gas Air Concentration

We reiterate that the swimming pool and the balance tank had been emptied on 31 August and 1 September 2011, respectively, and that the tank remained closed to external air for at least 60 hr since 3 September up to 5 September 2011 when the accident occurred.

#### 6.3.1 Service Rooms Adjacent to the Swimming Pool

The tank is 4.35 m long, 2.50 m wide, and has an average depth of 3.4 m (its floor being inclined) and a total volume of about 37 m^3^. On 6 September 2011, about 2 m^3^ of water occupied the balance tank bottom with a 12 to 38 cm depth (no presence of water was reported on the day of the accident). Two openings in a tank wall, 2.42 m above its bottom, correspond to the hubs carrying the pool water into the balance tank. In these hubs, a concentration of 120 and 550 ppm of H_2_S and of 1.0 and 1.8 vol.% of CO_2_ was measured on 6 September 2011, whereas 18 ppm of H_2_S and 4.4 vol.% of CO_2_ were found 20 cm above the water level (Site A).

**Figure 7:**
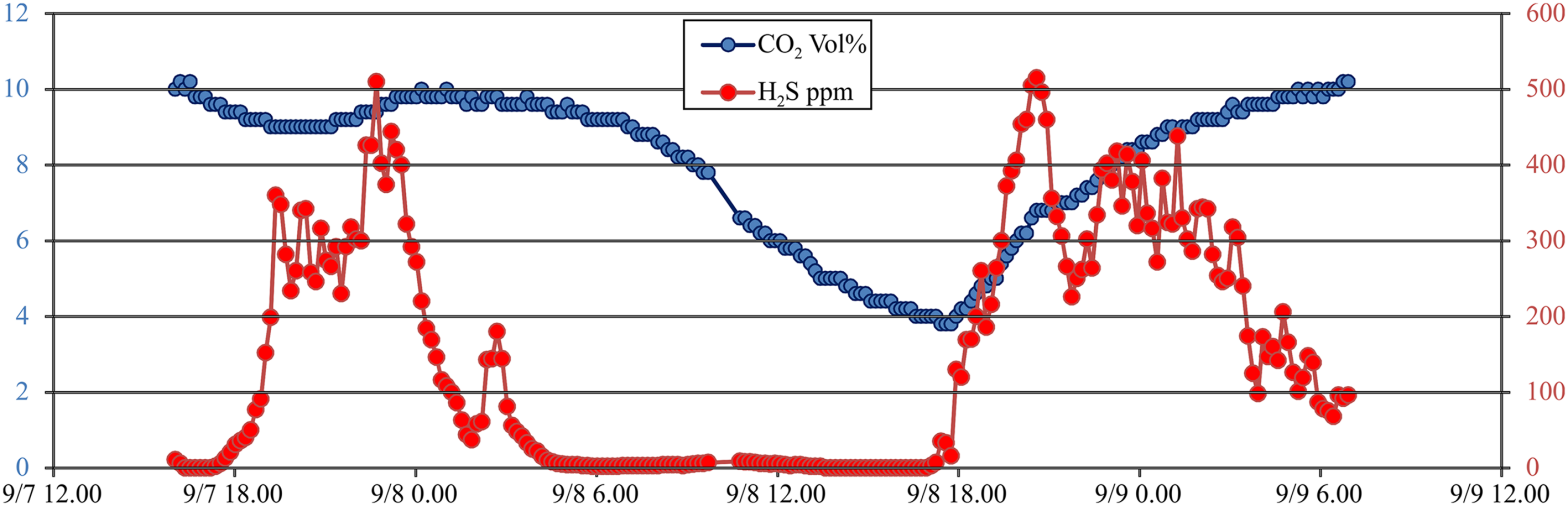
Continuous recording, from 7 to 9 September 2011, of CO_2_ and H_2_S air concentration at 20 cm above the water level in the bottom of the balance tank.

In order to reproduce the preaccident conditions, the balance tank was closed for 23 hr and on 7 September in Site A, the gas concentration had increased to 236 ppm of H_2_S and 10.6 vol.% of CO_2_. In the same Site A, at about 4:00 p.m. of 7 September, a Draeger device for continuous recording of gas air concentration was installed. When data were recovered on 12 September, we discovered that the device had functioned for only 50 hr, as acid gas had affected the metallic contacts of its electric feed. Recorded data are shown in Figure 7. Strong fluctuations were observed in the concentration of the two gases (4–10 vol.% for CO_2_ and 0–500 ppm for H_2_S), which however remained long at very dangerous levels (>8 vol.% for CO_2_, >400 ppm for H_2_S).

**Figure 8:**
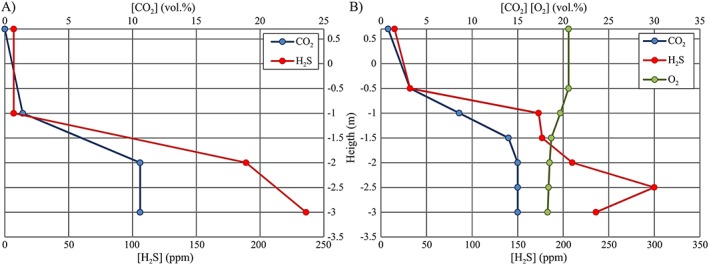
Air gas concentration profiles measured on 7 and 12 September in the balance tank (from ceiling to floor) (a) 23 hr and (b) 95 hr after the room closure.

On 7, 8, and 12 September, a vertical profile of gas concentration (including also O_2_ in the latter) was measured within the tank (from the ceiling to the floor) that had remained closed for 18 to 95 hr; results are reported in Table 3 and Figure 8. To be noted is the significant increase of CO_2_ concentration with depth; after 95 hr, it remained between 14 and 15 vol.% in the tank lower half where also H_2_S had hazardous concentrations (177–300 ppm) and O_2_ lowered at 18.5–18.3 vol.% (Figure 8b).

**Table 3:** CO_2_ (vol.%) and H_2_S (ppm) Concentration Vertical Profiles Carried Out in the Tank on 7, 8, and 12 September 2011. Time Within () Indicates the Hours Passed Since the Room Closure

Sites	7 (23 hr)		8 (18 hr)		12 (95 hr)		
	CO_2_	H_2_S	CO_2_	H_2_S	CO_2_	H_2_S	O_2_
Ground	0	9	0	5	‐	‐	‐
Tank rim	0	7	0.8	132	0.8	15	20.6
0.5 m under the rim	‐	‐	0.8	120	3.2	32	20.6
1 m under the rim	1.4	7	1.0	130	8.6	173	19.7
1.5 m under the rim	‐	‐	1.0	134	14.0	177	18.7
2 m under the rim	10.6	189	1.2	165	15.0	210	18.5
2.5 m under the rim	‐	‐	3.4	126	15.0	300	18.4
3 m under the rim	10.6	236	5.4	28	15.0	236	18.3

The following considerations can be made, remembering that before the accident of 5 September 2011, the balance tank had remained closed for at least 60 hr. Our data show that after the tank closure, the indoor air gas concentration increases rapidly. After 18 hr, high but not lethal concentrations were recorded (up to 5.4 vol.% of CO_2_ and 165 ppm of H_2_S; 8 September data; Table 3). After 23 hr, a concentration of 10.6 vol.% of CO_2_ and of about 200 ppm of H_2_S was reached at 170 cm from the tank bottom, a height corresponding to that of the opening of the upper respiratory tract of a standing person (7 September data; Table 3; Figure 8a). After 95 hr, CO_2_ air concentration was at lethal levels (14–15 vol.%) from 1.5 m below the rim to the tank bottom, with 177–300 ppm of H_2_S (potentially lethal if ≥250 ppm) and 18.7–18.3 vol.% of O_2_ (12 September data; Table 3; Figure 8b). Air concentration of CO_2_ and H_2_S increased with depth, but CO_2_ maintained a 15 vol.% value from 2 m below the tank rim to its bottom, and H_2_S showed a decrease in the lowest point (particularly on 8 September; Figure 8b), which likely reflects the different density, kinetics, and reactivity of the two gases.

**Figure 9:**
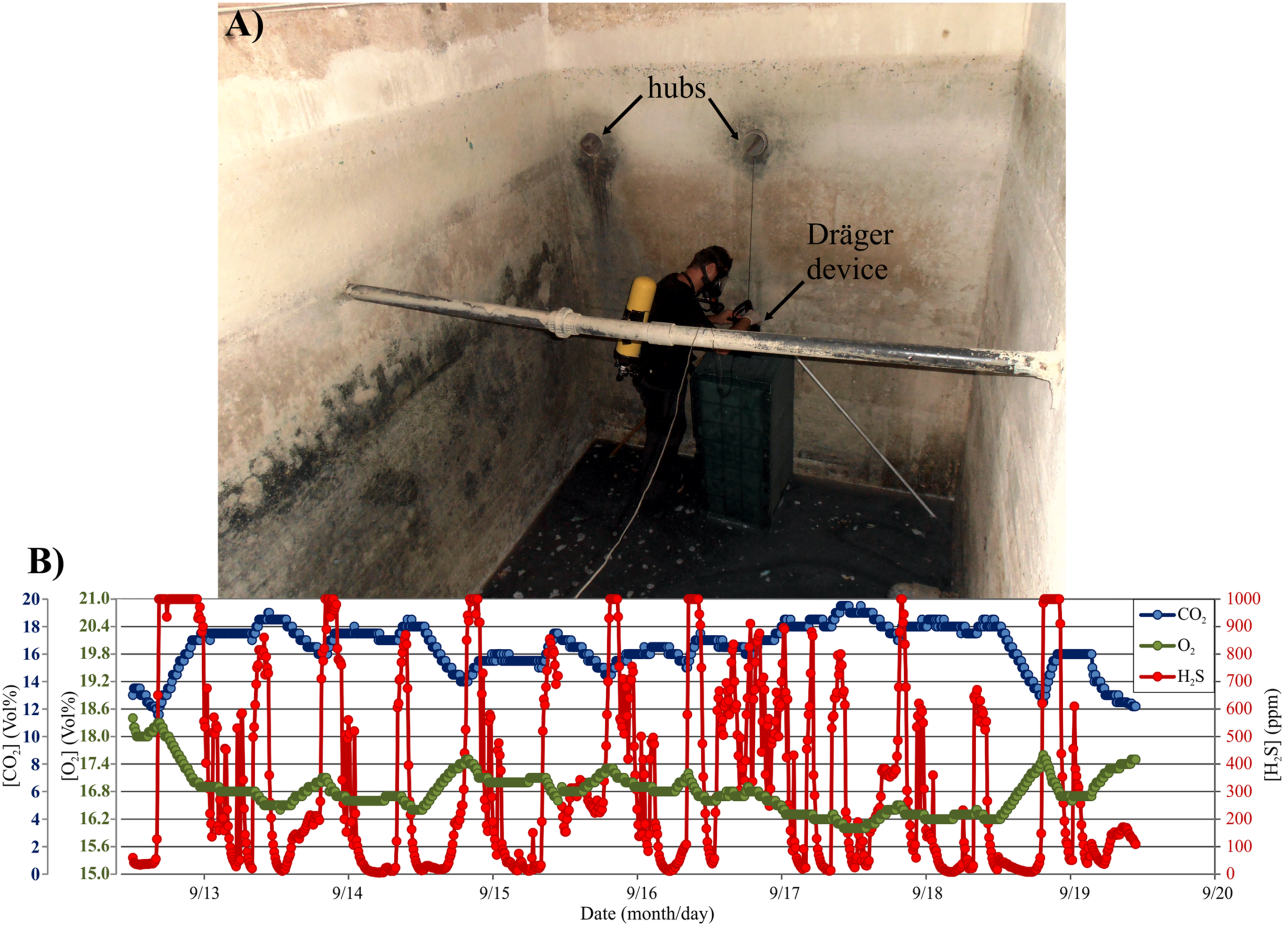
(a) The basement balance tank where the 5 September 2011 accident occurred. The location is indicated of the gas monitoring device, together with the two hubs connecting the tank with the swimming pool. (b) Results of the continuous monitoring of CO_2_, H_2_S, and O_2_ air concentration in the tank room, at 130 cm height, from 12 to 19 September 2011.

On 12 September 2011, a Draeger device was placed at 130 cm height from the tank floor in order to make a continuous recording for a week (one measure every 10′) of the CO_2_, H_2_S, and O_2_ air concentrations (Figure 9a). Results (Figure 9b) show that air CO_2_ concentration remained always above 12 vol.%, up to a maximum of 20 vol.%, and that H_2_S concentration had strong variations reaching frequently 1,000 ppm, the upper limit of the used device. The H_2_S variations were very rapid as the maximum value was reached in 30–60 min. It seems logical to infer that actual H_2_S concentration was significantly higher than 1,000 ppm, at least on 12 and 18 September when the recorded values remained at the full‐scale limit for 5 hr 50′ and 2 hr 30′ respectively. The O_2_ concentration was always below 18 vol.% and remained long at values between 16.8 and 16.2 vol.%, reaching a minimum of 16 vol.% on 17 September, when CO_2_ concentration was at its maximum (20 vol.%; Figure 9b). Processing of gas data by Fourier analysis shows periodical variations likely induced by atmospheric pressure, with typical 12 hr and 24 hr cycles (in Supporting Information Figure S1), as frequently observed (e.g., Laiolo et al., 2016; Rinaldi et al., 2012). Carbon dioxide and H_2_S have a direct correlation with atmospheric pressure (*r* = 0.36 and 0.26, respectively), and CO_2_ variation controls the O_2_ concentration (*r* = 0.96; see Figure S2); no correlation exists between atmospheric pressure and O_2_ (*r* = −0.06; data in Barberi et al., 2019).

In order to furtherly ascertain that most of the gas found inside the balance tank had been transported from the swimming pool through the two hubs, on 26 September 2011 the hubs were completely sealed and the gas was pumped outside from the tank until fresh air was restored. To ensure repeatability, the instrument was placed in the same place as the previous experiment. After nearly 3 days, the maximum concentrations of CO_2_ and H_2_S were respectively only of 1 vol.% and of 2 ppm, with O_2_ always above 20.4 vol.%. After 4 more days, maxima were 1.4 vol.% for CO_2_ and 3 ppm for H_2_S, with O_2_ > 20.4 vol.%. The results obtained indicate clearly that gas causing the 5 September accident had been transported into the tank from the hubs connecting it to the swimming pool. Therefore, particular attention had to be given to investigate the gas air concentration inside the pool space.

In the basement filter room, adjacent to that of the balance tank (see Figure 3a), all metallic components showed strong alteration by H_2_S, with sulfur incrustations. Here the indoor concentration was always above the short‐term exposure limit (STEL, see chapter 4) for both H_2_S and CO_2_ with maxima of 246 ppm and 4.2 vol.%, respectively.

#### 6.3.2 Swimming Pool

**Figure 10:**
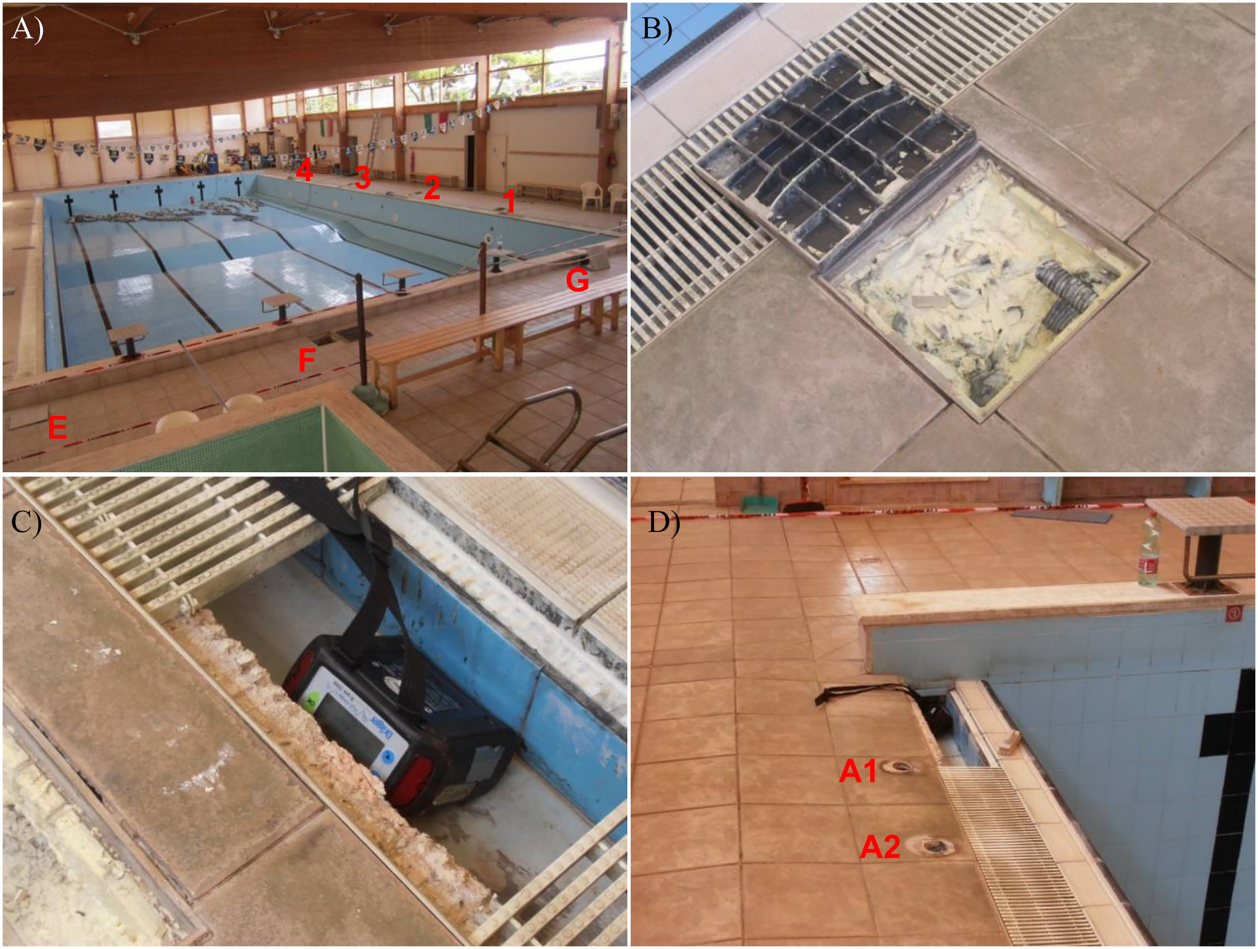
(a) The Lavinio Country Club swimming pool with the location of 1 to 4 light housings. (b) Light housing no. 1 with sulfur incrustations. (c) Device measuring gas air concentration placed into a lateral overflow channel. (d) Stair‐anchoring bores no. A1 and A2 and the nearby overflow channel. See Figure 3 for the precise location of these sites.

Around the swimming pool, air gas concentration was measured near the floor (at the nose level of bathing people) in the sites shown in Figure 3. In some light housings and in some parts of the small overflow channels, the presence of sulfur incrustations and of metal alteration by H_2_S was observed (Figure 10).

Results are reported in Table 4; note that when the CO_2_ concentration is indicated as higher than a given value (e.g., >6 vol.%), it means that registration was interrupted before reaching the real concentration value in order to avoid damages to the device, as H_2_S concentration was already at the device upper limit (1,000 ppm). On 26 September and 3 October, in order to measure the actual H_2_S concentration in the sites where the device had reached its upper limit, Draeger tubes were used. The very high concentrations of CO_2_ and H_2_S (up to 100 and 3.3 vol.%) indicate that most sites, particularly 1 to 4 and G light housings, but including almost all holes near the pool rim, release a huge quantity of gas that is conveyed by the lateral overflow channels into the balance tank. As a matter of fact, in the overflow channel near to 1–4 housings (location in Figure 3), H_2_S concentration was always >1,000 ppm with 19.5 to 21 vol.% of CO_2_; therefore, the concentration of both gases was at lethal levels.

**Table 4:** CO_2_ and H_2_S Air Concentration Near Ground Recorded Around the Swimming Pool (see Figure 3 for Site Location)

Site	6 Sept.		7 Sept. first series		7 Sept. second series		8 Sept.		19 Sept.		26 Sept.		3 Oct.	
	H_2_S ppm	CO_2_ vol.%	H_2_S ppm	CO_2_ vol.%	H_2_S ppm	CO_2_ vol.%	H_2_S ppm	CO_2_ vol.%	H_2_S ppm	CO_2_ vol.%	H_2_S Vol.%	CO_2_ vol.%	H_2_S vol.%	CO_2_ vol.%
A1	41	0.4	100	0.6	‐	‐	35	0	111	0	‐	‐	‐	‐
A2	251	4.6	322	7.6	‐	‐	89	4.8	115	0	‐	‐	‐	‐
1	450	0	>1,000	7	>1,000	7	>1,000	>9	>1,000	>2.8	2.4	58	1.3	98
2	374	2	11	0	>1,000	32	304	10.4	120	6	1.4	50	1.2	80
3	>1,000	92	>1,000	33	>1,000	32	>1,000	>8	>1,000	>42	3.3	96	2.4	100
4	>1,000	35	93	0.6	>1,000	82	>1,000	>6	60	0.8	1.8	80	2.5	100
B1	32	1	38	2.4	‐	‐	32	0	92	1.4	‐	‐	‐	‐
B2	65	1.2	220	1.2	‐	‐	32	0.8	76	1.6	‐	‐	‐	‐
C1	10	1.4	4	10.6	‐	‐	6	0	38	1.6	‐	‐	‐	‐
C2	7	1.8	5	6.8	‐	‐	4	0	26	3.4	‐	‐	‐	‐
5	7	1.2	5	0	4	1.2	4	0.5	54	0.6	‐	‐	‐	‐
6	7	17.5	2	0	0	7.4	5	0	19	0	‐	‐	‐	‐
7	3	0.8	0	0	0	9.4	4	0	20	0	‐	‐	‐	‐
8	5	1.8	0	1.6	0	6.8	4	0	15	1	‐	‐	‐	‐
D1	4	6.6	0	6.2	‐	‐	3	0	11	3	‐	‐	‐	‐
D2	4	2.8	0	1.4	‐	‐	2	0	10	1.4	‐	‐	‐	‐
E	354	1.8	0	0	7	1.6	3	0	10 (8^a^)	0 (0.6^a^)	‐	‐	‐	‐
F	>1,000	3.2	3	0	3	0.6	3	0	8 (7^a^)	0 (0^a^)	‐	‐	‐	‐
G	>1,000	22	7	0.4	>1,000	17	4 (>1,000^a^)	0 (13.5^a^)	10 (>1,000^a^)	0 (2.4^a^)	0.7	27	‐	‐
H	20	0.4	6	0	‐	‐	4	0	16	0	‐	‐	‐	‐

*Note*. 6 Sept. measures with housings opened since 10′. 7 Sept.; first series, measures with closed housings; second series, measures immediately after their opening; 8 and 19 Sept.: measures with closed housings; and 26 Sept. and 3 Oct.: measures with Draeger tubes after housing opening.

Abbreviation: ppm, parts per million.

a Measures after opening.

In the years before the 2011 accident, some swimmers presented complaints reporting erythema and conjunctivitis, and some families cancelled the pool subscription for their children. However, no formal complaint was presented to health and safety officers.

Finally, in the children's bathroom and shower room (Figure 3), an H_2_S concentration of 100–185 ppm with 0.8–1.0 vol.% of CO_2_ was found with strong evidences of metal alteration by H_2_S.

## 7 Discussion and Conclusion

The accident that occurred at LCC in September 2011 increases the number of lethal accidents caused by endogenous gases in volcanic and geothermal areas (IVHHN, 2005).

Many geoscientific evidences coherently indicate that LCC facilities have been built in a site severely exposed to the hazard of cold endogenous gas emission. These include:

- High CO_2_ concentration in the soil (23 vol.% at 50 cm depth).
- Strong release from a 42 m deep well (TCL1) of a gas with a composition (CO_2_ = 95.8 vol.%, H_2_S = 4.2 vol.%, and ^3^He/^4^He = 0.65) similar to that of Tor Caldara, the nearest discharge site of endogenous gas.
- Gas blowout occurred in the 1970s years during drilling of TCL0 well, indicating the presence at shallow depth of a gas pressurized aquifer.
- High soil CO_2_ and H_2_S flux, up to 898 and 7.155 g·m^−2^·day^−1^, respectively, measured in the zone of TCL0 well and probably generated by the well drilling that increased the permeability of the soil to the rising gas, as it has been observed in several other similar cases (Carapezza et al., 2019).

Under such geological conditions, it is easy to infer that during the excavations made to build the swimming pool in the years 1988 and 1989, the conditions have been created allowing gas to reach the surface from the subsoil. As a matter of fact, we found high and frequently lethal concentrations of both CO_2_ and H_2_S in all the small empty spaces around the pool rim, as light‐housings and pool stair‐anchoring holes. It seems logical to infer also that over time the gas opened cracks in the concrete of these channels and holes.

Our study shows that the accident of 5 September 2011 occurred because the men who descended into the emptied water balance tank inhaled a nearly lethal mixture of oxygen‐poor air, carbon dioxide, and hydrogen sulfide. Results of the experiments, carried out with the aim of reproducing the accident conditions, showed that:

- The gas had been transported from the swimming pool into the emptied balance tank through two hubs, usually carrying the pool overflow water, as no appreciable gas increase has been recorded in the tank atmosphere, after having totally sealed the two hubs for several days.
- The reported time (at least 60 hr) during which the balance tank had remained closed to the external air before the accident, had been certainly long enough to reach nearly lethal indoor conditions.

The air CO_2_ and H_2_S concentration indoor the balance tank, at the human breathing height, was at the moment of the accident likely around 14–20 vol.% and 250–400 ppm, respectively, with an oxygen air content of 16.0–17.5 vol.%. As the site is at standard temperature and pressure conditions, both gases are denser than air and they accumulated near the room floor; therefore, after unconsciousness, the men inhaled a more gas‐rich and oxygen‐poor air mixture. We recall that during 50 hr of recording, gas concentration remained long at very dangerous levels (CO_2_ = 8–10 vol.%; H_2_S = 400‐500 ppm) at about 30 cm from the tank bottom (Figure 7). According to all medical reports, we would expect the effects of CO_2_ to wear off on the return of the victims to fresh air and for them to eventually make a good recovery (IVHHN, 2005 and references therein). Exposure to dangerously raised level of H_2_S in the air is more likely to be fatal in gas accidents, being much more toxic than CO_2_ and usually requires emergency resuscitation to survive. Brain injury is due to lack of oxygen in long periods of unconsciousness.

The fate of the different persons depended on the order in which they descended into the tank and then on the time they passed into this gas chamber.

The swimming pool was shut down on 5 September 2011. It was reopened in late October 2012, after the completion of precautionary measures aimed at reducing the gas hazard. These include 1) the excavation of a large fenced channel intercepting the old TCL0 gas leaking well and aimed at dissipating the emitted gas into the atmosphere; 2) permanent access interdiction into all basement rooms; 3) the floors of children bath and shower rooms (where CO_2_ and H_2_S air concentration up to 1 vol.% and 185 ppm, respectively, had been measured) were raised by 20 cm, living a ventilated crawl space, equipped with a forced air circulation system, and covered by a plastic damp roof membrane; 4) installation of a forced air ventilation system in the pool space; and 5) daily check of CO_2_ and H_2_S concentration in air by the staff personnel in all rooms (a permanent gas monitoring system with alarm was initially recommended but due to the many difficulties encountered in its maintenance, it was replaced by a discrete monitoring, in agreement with the public officer). Moreover, public officers check the safety conditions of LCC at least twice per year.

In 2018 the owner and manager of LCC were convicted by a civil trial to a compensation for the families of the persons involved into the accident (presently under appeal). The penal trial has not yet ended.

To be noted that in late 2010, we completed for the Civil Protection of Regione Lazio a detailed multiyear study of the wide Rome Metropolitan City, where all areas exposed to gas hazard had been identified. In January 2011, Regione Lazio distributed the report to all interested municipalities asking them to adopt specific prevention measures. Anzio municipality (which includes Lavinio) received this report but no action was undertaken before the 5 September 2011 accident. This question is debated in the still ongoing penal trial where Anzio municipality is among the defendants. Soon after the accident, Anzio municipality recommended the adoption of precautionary measures for all basement rooms present within the areas exposed to gas hazard. In November 2011, we presented to Regione Lazio–Direzione Regionale Ambiente, a scientific study including the limits of all areas exposed to gas hazard within the region and suggesting technical and methodological guidelines for updating the investigations required in urban planning (INGV, 2011). On January 2012, a regional decree (N. A00271 19/01/2012) was issued introducing obligation to carry out geochemical investigations (soil CO_2_ flux and concentration) for land use planning and establishing the following rules: areas with soil CO_2_ concentration < 1 vol.% are considered suitable to host buildings; those with CO_2_ concentration between 1–2 vol.% and 2–5 vol.% requires the adoption in building design of specific technical prescriptions to reduce gas hazard; and areas with soil CO_2_ concentration > 5 vol.% are classified as no building areas. These measures reduce the risk of future accident; however, no prevention measures have been taken to protect people living in houses already built in hazardous gas emitting zones. Here, ventilation systems should have been recommended for basement and ground floor rooms, as well as permanent gas monitoring systems with alarm in public buildings, together with periodic campaigns aimed at increasing the risk awareness of the resident population. Authors suggested these precautionary measures in all the reports presented to Regione Lazio.

## Conflict of Interest

The authors declare no conflicts of interest relevant to this study.

## Supporting information



Supporting Information S1
