# Supplementary material for: Anomalous Discharge of Endogenous Gas at Lavinio (Rome, Italy) and the Lethal Accident of 5 September 2011
Source: Geohealth. 2019 Dec 3;3(12):407–22. doi: 10.1029/2019GH000211 (PMC7038904; doi:10.1029/2019GH000211)
Supplement: Supplementary file 1 — Supporting Information S1 [file GH2-3-407-s001.pdf]

AGU PUBLICATIONS

GeoHealth

Supporting Information for

Anomalous Discharge of Endogenous Gas at Lavinio (Rome. Italy) and the Lethal Accident of 5 September 2011

F. Barberi[1], M. L. Carapezza[1*], L. Tarchini[1], M. Ranaldi[1], T. Ricci[1], A. Gattuso[2]

[1] INGV – Istituto Nazionale di Geofisica e Vulcanologia, Sezione Roma1, Rome, Italy

[2] INGV – Istituto Nazionale di Geofisica e Vulcanologia, Sezione di Palermo, Italy

*Corresponding author: Maria Luisa Carapezza (marialuisa.carapezza@ingv.it)

Contents of this file

Figures S1 and S2

Introduction

Figure S1 shows the periodograms of atmospheric pressure and of CO_2. H_2S and O_2 concentrations elaborated by Fourier analysis on the indoor continuous monitoring dataset from 12 to 19 September 2011.
Figure S2 shows the variation of the atmospheric pressure and of CO_2, H_2S, O_2 concentrations continuously measured at 130 cm height in the balance tank from 12 to 19 September 2011 (average concentration values recorded every 10’ and every 30’ for atmospheric pressure). These figures help in interpreting results presented in Figure 9 and in the discussion and conclusion.

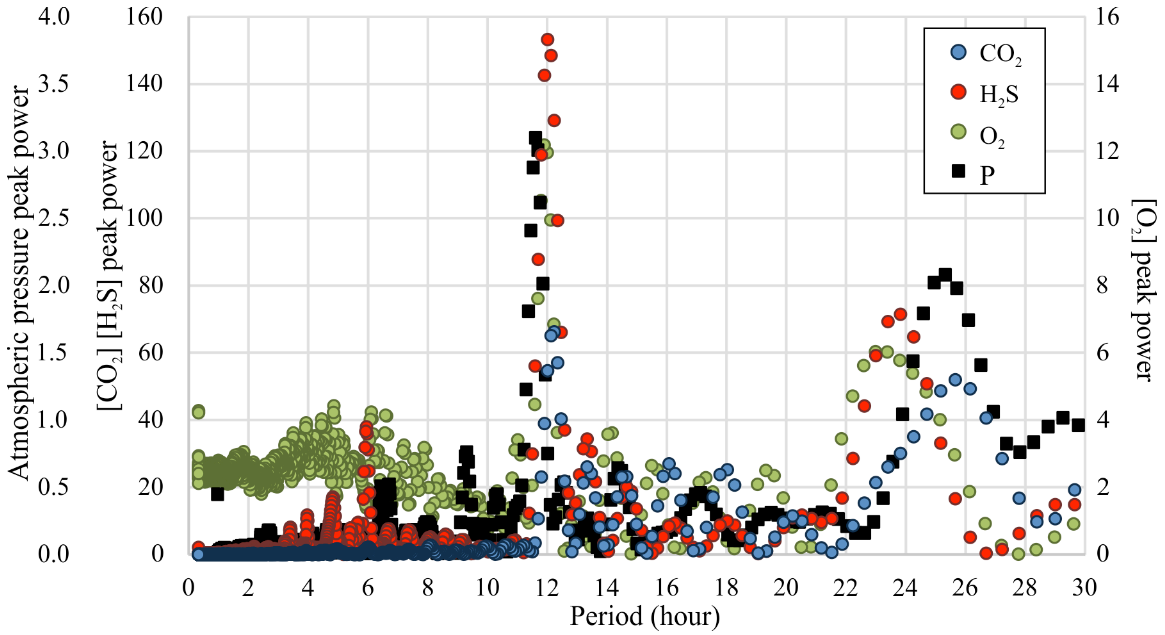

Figure S1. Periodograms of the atmospheric pressure (black dots) and of the CO_2 (blue dots), H_2S (red dots), O_2 (green dots) concentrations continuously measured from 12 to 19 September 2011, at 130 cm height in the balance tank (average concentration values recorded every 10' and every 30' for atmospheric pression). Peak power is proportional to the square of sinusoid amplitudes.

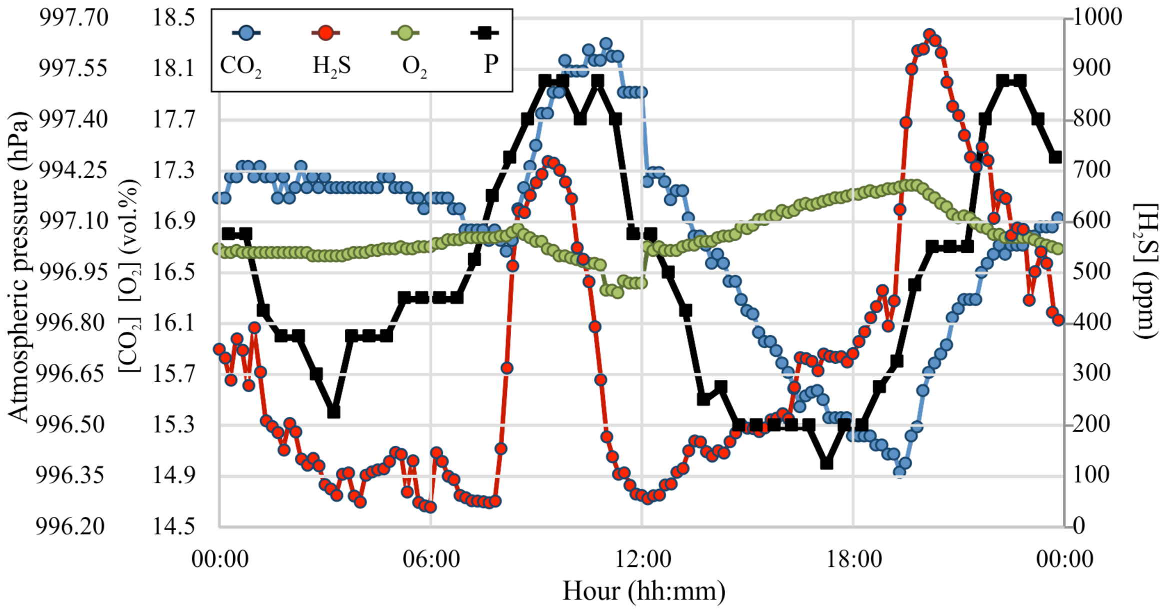

Figure S2. Variation of the atmospheric pressure (black dots) and of CO_2 (blue dots), H_2S (red dots), O_2 (green dots) concentrations continuously measured at 130 cm height in the balance tank from 12 to 19 September 2011 (average concentration values recorded every 10' and every 30' for atmospheric pressure).
